# When species don’t move together: Non-concurrent range shifts in Eastern Pacific kelp forest communities

**DOI:** 10.1371/journal.pone.0303536

**Published:** 2024-05-24

**Authors:** Mary R. Cortese, Amy L. Freestone

**Affiliations:** Department of Biology, Temple University, Philadelphia, Pennsylvania, United States of America; Universidade dos Acores, PORTUGAL

## Abstract

Species range shifts due to changing ocean conditions are occurring around the world. As species move, they build new interaction networks as they shift from or into new ecological communities. Typically, species ranges are modeled individually, but biotic interactions have been shown to be important to creating more realistic modeling outputs for species. To understand the importance of consumer interactions in Eastern Pacific kelp forest species distributions, we used a Maxent framework to model a key foundation species, giant kelp (*Macrocystis pyrifera)*, and a dominant herbivore, purple sea urchins (*Strongylocentrotus purpuratus)*. With neither species having previously been modeled in the Eastern Pacific, we found evidence for *M*. *pyrifera* expansion in the northern section of its range, with no projected contraction at the southern range edge. Despite its known co-occurrence with *M*. *pyrifera*, models of *S*. *purpuratus* showed a non-concurrent southern range contraction and a co-occurring northern range expansion. While the co-occurring shifts may lead to increased spatial competition for suitable substrate, this non-concurrent contraction could result in community wide impacts such as herbivore release, tropicalization, or ecosystem restructuring.

## Introduction

As scientists grapple with the intricate system-wide impacts of climate change, it is becoming clear that impacts scale far beyond habitat suitability. We know that with changing climate comes changing distributions [1]. In ocean systems, changing temperatures are leading to a large-scale poleward shift in many organisms’ natural geographic ranges. Despite being understudied compared to terrestrial systems, marine range shifts are clearly tracking with climate change and are occurring at faster rates than in terrestrial systems [2–5]. While species are commonly considered to be more vulnerable at a warmer lower latitude range edge, it has been shown that vulnerability to climate is consistent throughout a species range [6] raising questions about how these range shifts will impact species across their range.

Although temperature strongly influences species range, biotic factors like species interactions are also known drivers of range boundaries [7–9]. As species seek refuge from warming, we have little knowledge on how biotic interactions in combination with warming will impact range boundaries. Despite calls for the inclusion of biotic interactions into range models [10], such studies are rare. Those that do include biotic interactions are mostly on terrestrial systems with species with small home ranges [7] leaving marine organisms with broad ranges as largely understudied. Distribution modeling has become the predominant method for analyzing potential range shifts, but models based only on abiotic factors are thought to mask species vulnerability [11, 12], revealing that changes in distribution may be a consequence of altered species interactions in addition to the direct impacts of climate change [10, 13].

There is evidence that as ranges shift, movement of separate species will create ecological mismatches altering community composition and abundance [14, 15] leading to reshaped trophic dynamics. Mismatched range movement has caused the restructuring of plankton communities with subsequent impacts on higher trophic level grazers [16]. Altered ranges of herbivorous marine fish have led to reduced macroalgal cover in Japan and the Mediterranean—transforming ecosystems from kelp canopy to rocky barrens [17]. In terrestrial systems, plant communities with historically low consumer pressure have been shown to be at risk of decline due to herbivore range shifts into the system [18]. Additionally, changing composition in marine environments can have impacts for coastal zone management of industries such as fisheries, tourism, and even human health [19]. Despite beginning to understand the scale and impacts of range shifts across systems, questions remain regarding which biotic interactions contribute the most to determining new range boundaries, and the subsequent impact these changes have on shaping emergent communities [13, 20].

**Fig 1:**
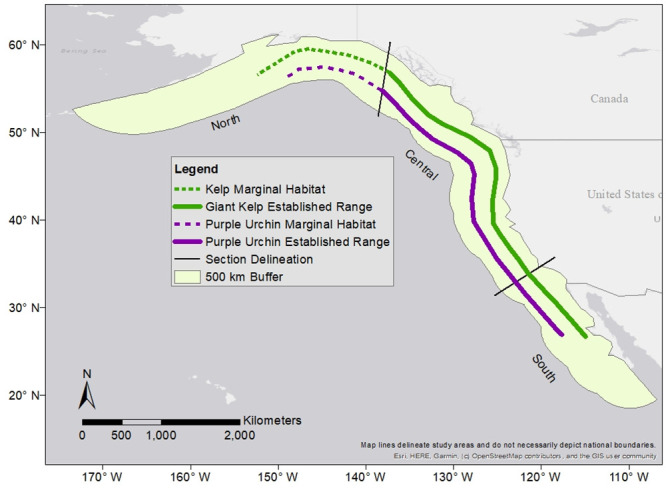
Current species ranges. Current ranges of giant kelp and purple urchin across the Eastern Pacific Ocean with bounded study area containing 500 km buffer from the coast (N 19.5277594–61.064468, W 173.391988–106.863603). Section delineation lines mark where range was split into northern, central, and southern sections. Dotted lines indicated marginal habitat where there are known occurrences but non-continuous populations.

Kelp forests, one of the most productive marine ecosystems in the world [21], and their urchin herbivores, span the Northeast Pacific Ocean from Alaska to Baja California. Kelp forests serve as both an iconic temperate marine ecosystem of conservation importance and an ideal model for understanding plant-herbivore range dynamics under climate change. The importance of these ecosystems elicited the creation of long term monitoring programs [22] and marine protected areas throughtout the Eastern Pacific [23, 24]. In the southern range, giant kelp (*Macrocystis pyrifera* [Linnaeus], C.Agardh, 1820) forms the foundation of the forest, with purple sea urchins (*Strongylocentrotus purpuratus*, Stimpson, 1857) as the dominant herbivore (Fig 1). Purple urchins range from Isla Cedros, Baja California, MX (~28 degrees N) to Cook Inlet, Alaska, USA (~58 degrees N, [25]) although more constrained range limits have been published more recently [26]. Giant kelp spans from Punta Hipolito, Baja California, MX (~27 degrees N, [27]) to its most northern observations in Kodiak Island, Alaska, USA (~60 degrees N, [28], Fig 1). Purple urchins have a broad diet, but have shown a preference for giant kelp when available [29] and are closely paired with giant kelp across their range [30]. Through their grazing, urchins can drive phase shifts between healthy kelp forests and overgrazed barren ecosystems [31, 32], reshaping whole communities. Across sections of California, USA large swaths of kelp forest have been converted to urchin barrens, reducing once flourishing ecosystems into a rocky patchwork [33]. In addition to herbivory, purple urchins are important for bioerosion [26] and nutrient cycling [34], as well as support an established fishery.

Physiology and species interactions in these kelp forests have been widely studied in the northern part of their range, from Alaska to California, but the southern edge is comparatively understudied, despite evidence that this section will be most sensitive to climate warming induced community change [35]. *Macrocystis pyrifera* has its own history of climate induced range contraction in southern California and other populations globally [36, 37]. Models of species sensitivity to climate change indicated that canopy forming kelps, including *Macrocystis*, would experience declines in survival rate with increasing temperatures [38]. Temperature in particular has been noted as an important limiting factor on the southern range edge of kelp, while other factors, such as light availability, may be important on the northern edge [39]. During short-term warming events, British Columbia saw canopy loss for multiple canopy forming kelp species [40]. Short-term warming in Baja California led to significant changes in kelp-bed structure including a decrease in temperate species and an increase in tropical species abundance [41]. Notably, all echinoderms showed reductions in abundance except for the crowned sea urchin, *Centrostephanus coronatus* [41, 42]. Shifts in urchin abundance are known to drive community change in kelp forest ecosystems [43], but no distribution model has been attempted for purple urchins despite its known ecological and economic importance and potential for climate induced geographic change.

More broadly, there is a surprising lack of research on the response of benthic marine invertebrates to climate change [4], even though they are critical to the functioning of marine ecosystems. Warming will impact ectothermic species more than self-regulating endotherms [44] and is also expected to be more intense for marine ecotherms than for terrestrial species [45]. Marine ectotherms have been shown to demonstrate movement on both ends of their range, as opposed to endotherms who predominately experience northern edge expansion events [20]. Sea urchins have undergone range shifts in other populations around the world [46], but the projections have never been assessed for Eastern Pacific species or alongside projections of their prefered food source. Other species of urchins have a life history closely linked to thermal conditions [47], with thermal stress as a known cause of increased mortality [48], but these important metabolic thresholds have yet to be assessed for purple urchins. We know that many urchin species typically live near the top of their thermal limit [49–51] indicating that continued thermal change could have large impacts on habitat suitability [49]. Interactions between purple urchins and giant kelp have been studied in relation to trophic networks and kelp forest-urchin barren alternative stable states [52–54], but only one study has assessed this interaction under climate change conditions [55], and none have attempted to assess changing occurance patterns geographically across the entire species range.

We used a species distribution modeling approach to understand both current day range limits and projected limits under climate change scenarios of the purple urchin and its primary resource, giant kelp. We tested the hypothesis that giant kelp will undergo a southern range contraction leading to a concurrent contraction in purple urchins due to its thermal vulnerability and preference for giant kelp. Additionally, the inclusion of kelp presence as a biotic variable in models of urchin distribution will lead to more pronounced contractions than modeling of purple urchins using abiotic variables alone, demonstrating the value of modeling interacting species together. Lastly, we tested the hypothesis that purple urchins would undergo a northern edge expansion due to the weakening of temperature as a biogeographic barrier and the availability of giant kelp as a food source to facilitate expansion. We expect giant kelp populations to remain stable on the northern range edge due to the stability of other environmental barriers, like light availability, that may limit kelp expansion despite changing temperatures. These models seek to understand potential changes to large scale occurrence patterns across an ecologically important ecosystem, giving management, fisheries, and scientists the opportunity to better prepare for the impacts of global change across the region.

## Materials and methods

To model the distribution of giant kelp and purple urchins we used Maximum Entropy Modeling (Maxent; [56]), an open-source java-based species distribution model (SDM). Maxent was chosen over other modeling platforms due to its ability to handle presence only data and highly correlated predictor variables which are common in ecological systems [57]. We used maxent to predict the probability of occurrence (0–1) of each focal species (cloglog output format) across three sets of models. The cloglog output format was selected over other output options due to its ease of interpretation and applicability for our modeling goals [57, 58]. We first modeled the distribution of kelp using kelp presence data and relevant environmental predictor variables (hereafter, kelp model). Similarly, we modeled urchin distribution using urchin presence data and relevant environmental predictors (hereafter, urchin model). We then modeled the distribution of urchins, using urchin presence and environmental predictors, along with kelp presence as an additional predictor variable (hereafter, urchin + kelp model). In this urchin + kelp model, continuous probability outputs from our kelp model were rasterized and included along with other environmental variables. This type of sequential approach was shown to improve distribution models in other species [59]. This approach allowed us to understand potential range shifts for both species as well as to evaluate the benefit of modeling interacting species within the same predictive framework.

To forecast distributional shifts due to climate change, each set of models was run for ambient temperatures, and three climate scenarios (relative concentration pathways [RCP] 4.5, 6.0, and 8.5) at two time points (2050 and 2100) based on IPCC climate projections [60]. While other lower emissions scenarios exist, RCP 4.5 is the lowest scenario without strict emissions mitigation. Because of the uncertainty related to achieving necessary mitigation, we decided to use 4.5 as our lowest emissions scenario. The modeled kelp distribution for each scenario was input into each respective urchin + kelp model. We defined the study area using the spatial extent of available species occurrences in the Northeast Pacific Ocean to inform a maximum and minimum range extent (S1 & S2 Figs in S1 File). We then restricted the model to within 500km of the range edges and the coast to capture all potential habitat (Fig 1). Occurrence observations outside of those geographic parameters were considered outliers and excluded from the study. Data were cropped to this study area using ArcMap Desktop version 10.8 [61]. For each model output, species range was calculated as well as the area of range gained and lost in each climate scenario when compared to the current distribution. Additionally, the area of range was calculated for each range edge by dividing the range into a northern, central, and southern sections (Fig 1), based on the Marine Ecoregions of the World [62] as well as known biogeographic barriers within the species range (Point Conception, California, USA), to better understand the directionality of range shifts at different climate zones. Sub-sectioning of data was also done using ArcMap Desktop, and all maps were produced using ESRI basemaps [63].

### Occurrence data

Presence data was acquired for *M*. *pyrifera* using the Global Biodiversity Information Facility database (n = 1,824, [64]). Presence data for *S*. *purpuratus* was acquired from public databases including the Global Biodiversity Information Facility database (n = 2,389, [65]), Aquamaps (n = 116, [66]), Integrated Digitized Biocollections (n = 58, [67]), Australia’s Integrated Marine Observing System (IMOS) via the Australian Open Data Network (n = 84, [68–70]), and Ocean Biodiversity Information System (n = 112, [71]). Records were also included from relevant literature ([30], n = 4, [72], n = 1, [73], n = 9, [74], n = 2). Occurrence records are shown in S1 & S2 Figs of S1 File. For citizen science data included in GBIF observations, only research grade observations were used, meaning species identification were verified by at least two independent users and the observation included a photo, georeferenced location, and date.

Due to the aggregate nature of open-source data, occurrences can be biased towards areas with high human populations, rather than evenly distributed across a species range [57]. To account for this bias within our models we created two bias files, one for each species model, with data collected from the Global Biodiversity Information Facility database [75, 76]. This approach assumes that observers who identify a closely related species would have also identified the species of interest in the same location if it had been present, reducing pseudo-absences to a more accurate range. This kind of target-group background bias assessment improves average model performance and predicted distributions [77]. Each bias file was comprised of a raster with the approximate survey effort across the range. This survey effort was estimated by the distribution of occurrence records of closely related species. For the kelp bias file, we approximated effort by collecting records of the phylum *Ochrophyta*, the phylum encompassing all brown algae, across the study area (n = 171,061). For the purple urchin bias file, we approximated effort by collecting records of the phylum *Echinodermata*, the phylum encompassing sea stars, urchins, and sea cucumbers, across the study area (n = 31,577).

### Environmental variables

To model the abiotic environment of the focal species, we selected physical and climatic variables that are ecologically relevant and known or expected to limit the distribution of either *M*. *pyrifera* or *S*. *purpuratus* (S1 Table in S1 File). While it is common for correlated variables to be removed from models, Maxent uses a machine learning approach when dealing with variable correlation. This distinction allows for the inclusion of all relevant variables, despite potential correlation, and for the model to determine which variables are most important [78]. Therefore, all relevant variables were retained in the model. Physical seafloor features were acquired from MARSPEC [79] and included concavity, depth, plan curvature, profile curvature, and slope (S1 Table in S1 File). These data represent current day conditions at a 30 arcsec resolution (approximately 1km at the equator) and clipped to the study area. Other environmental data for current day and each predicted RCP scenario were gathered from Bio-Oracle [80, 81] version 2.1 at a spatial resolution of 5 arcmin (approximately 9.2 km at the equator) and interpolated to fit the 1 km scale of the other variables. For Bio-Oracle data, current day values correspond to a long-term average from 2000–2014 across the benthic average depth of each pixel. The mid-century timepoint corresponds to data across the benthic average depth from 2040–2050. The end of century timepoint corresponds to data across the benthic average depth from 2090–2100. Primary production and light at bottom were taken as averages for current day. Salinity and temperature were included with three variables (maximum, minimum, and mean) for current day and each climate scenario. Current velocity was also included as two variables (maximum and minimum) for current day and each climate scenario.

### Species distribution modeling

Maxent was parameterized based on considerations from Merow et al. [78]. Maximum iterations were increased from 500 to 5,000 to allow for adequate time for model convergence, and a random seed that was used to ensure replicate independence. Bias files (as described above) were included to account for sampling bias and to confine background data. Regularization multiplier was kept at the default of 1 due to the ability of this value to perform well across a variety of taxonomic groups [78]. For each model we randomly withheld 75% of the occurrence data to be used as a training dataset. The remaining 25% was used in each test run. 10,000 background points were randomly selected to represent ambient environmental conditions in each model, and all samples were added to the background data pool from which selection occurred. For each model type (kelp, urchin, urchin + kelp), 30 bootstrapped replicates were run for each climate scenario (current day and RCP 4.5, 6.0, and 8.5) and timeframe (2050 and 2100) producing one output map per replicate as well as an average. A jackknife analysis was run to assess variable contribution to model outputs. Model fit was evaluated using an area under curve (AUC) value where 0.5 is a prediction no better than random chance, and 1 is a perfect prediction. Test omission rates were included as a threshold dependent evaluation metric for two thresholds, the minimum training presence and 10^th^ percentile training presence. We compared these values to the expected theoretical values of zero and 10% (0.10) respectively [82]. Values higher than these indicate possible overfitting.

### Output processing

To evaluate the models beyond visual assessment, Maxent outputs were processed to quantify the area of occupied space and the amount of range gained and lost under each scenario. To understand directionality of range shifts, differences in model outputs were tested for the north, central, and southern range sections (Fig 1). The 30 replicates from each model scenario were split into groups of 10. Each group of 10 was assigned to a range section (north, central, and south) and cropped down to that section of the range. Each range section was then processed to calculate the area of occurrence using the predicted suitability threshold of 0.05 corresponding to a 5% chance of occurrence. This threshold corresponds to the average minimum training presence threshold across all species and model runs. By using the average minimum training presence, we ensured all of our training data would be included in our model outputs. This approach provided a conservative estimate of range size and allowed for the inclusion of areas with highly varied occurrence patterns [11, 56]. Pixels that had occurrence levels that fit that criterium were summed to give a value for the predicted range.

For current day models, the predicted range was the summation of suitable pixels at current day. Area that was gained under a climate scenario represented pixels with less than 5% chance of occurrence in the current day model and that exceeded 5% under that climate scenario. Similarly, area lost included pixels with occurrence over 5% in the current day model and that dropped below 5% under that scenario [11]. Range calculations were done using published code [11] in R version 4.2.2 [83] using the packages *raster* [84] and *sp* [85].

### Data analysis

To test the hypothesis that kelp and urchins will undergo range shifts under climate scenarios, we ran four generalized linear models (GLM). Each time point was modeled separately, rendering two urchin models (2050 and 2100), and two kelp models (2050 and 2100). Each model included the predicted range as the response variable, and section (north, central, south), scenario (current, 4.5, 6.0, 8.5), and the interaction of scenario and section as the predictor variables. In addition, urchin models included species (urchin only and urchin + kelp) as a predictor variable and all interactions between species and other factors. Each model was run with a gaussian distribution. No other user specified parameters were modified. Diagnostics were run prior to model fitting to check model assumptions including for normality and homogeneity of variance. Differences among variable levels were tested with Tukey all means comparisons. GLMs were run in R version 4.2.2 [83] using the packages *dyplr* [86] for data preparation, *glmmTMB* [87] for the linear models, *MuMIn* [88] to extract *R*^2^ values, *stats* [83] for Tukey comparisons and *ggplot2* [89] and *wesanderson* [90] for visualizations.

## Results

**Fig 2:**
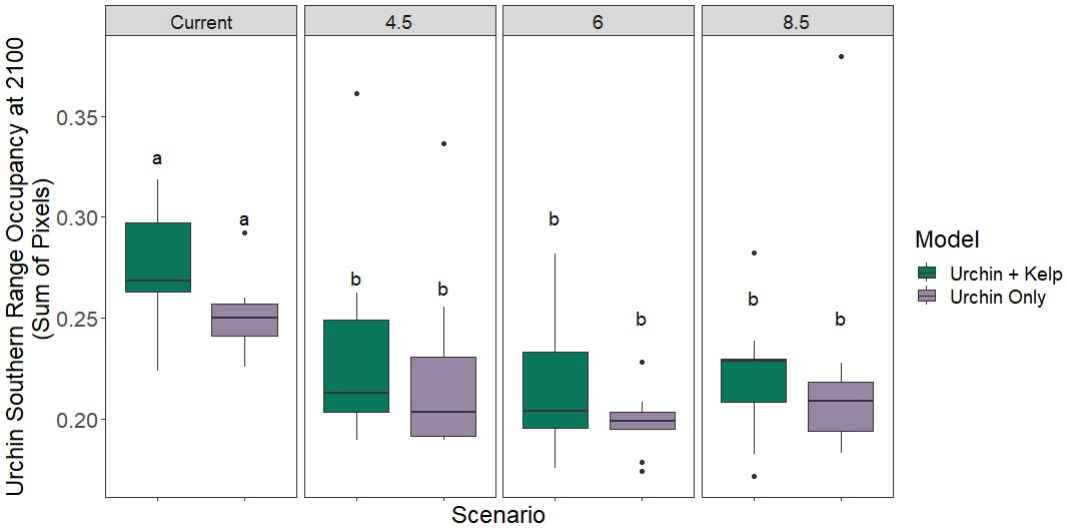
Urchin southern range occupancy. Urchins occupied less of the southern range edge under all climate change scenarios by 2100 than at current day (Scenario × Section Tukey p-values = 0.04, 1.2E-5, and 0.005 for each RCP respectively; S2 Table in S1 File). See supplement (S3 Fig in S1 File) for mid-century predictions.

**Fig 3:**
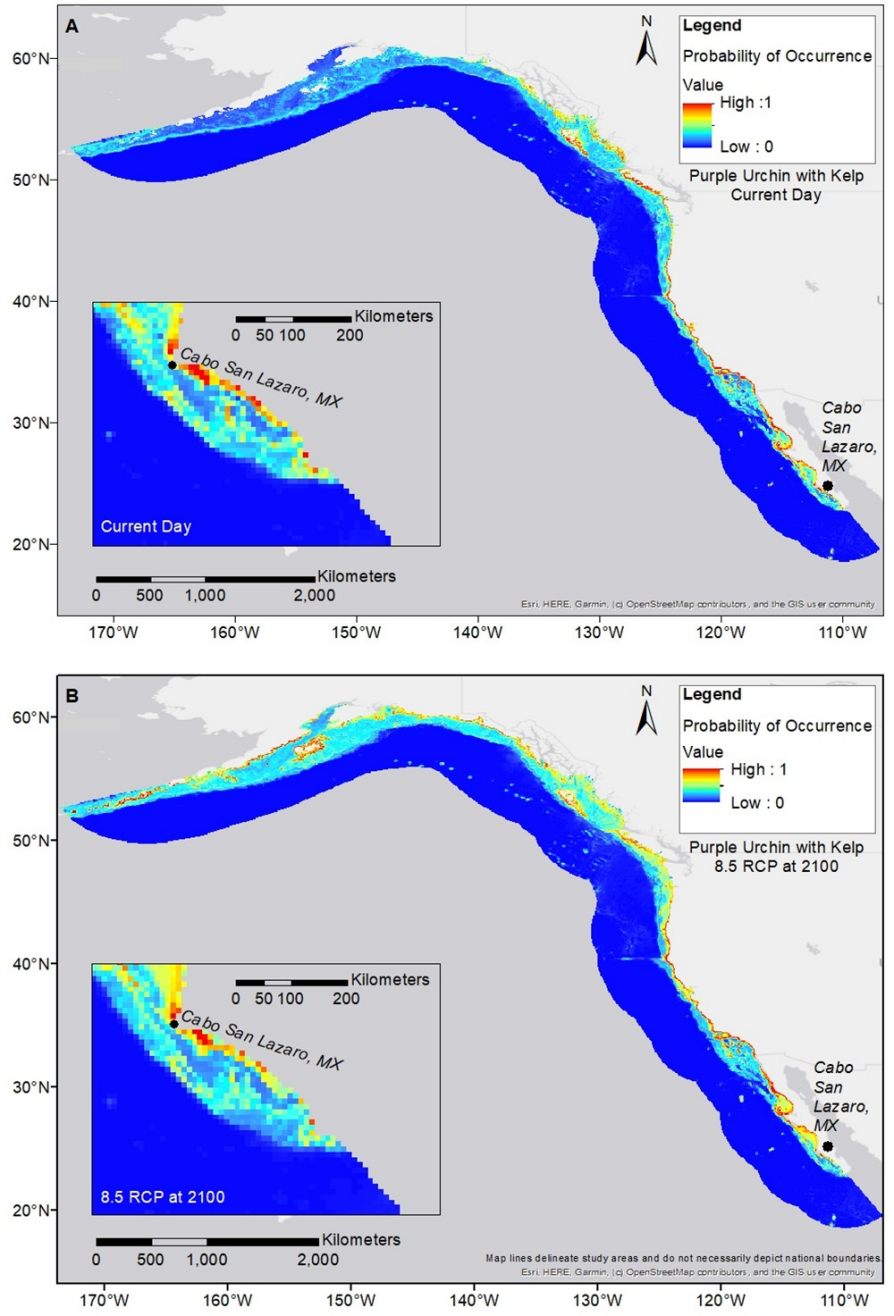
Purple urchin distribution maps. Purple urchin is modeled with giant kelp at RCP 8.5 for end of century; (a) shows the current day projection; (b) shows the RCP 8.5 scenario for 2100. Pullouts focus on the far southern edge of the species range. Visual reductions in geographic spread are seen at the farthest southern edge by end of century.

Under climate change scenarios, purple urchin and giant kelp are predicted to have non-concurrent range shifts in the southern portion of their range, with a contraction occurring for purple urchin populations but not for giant kelp. Purple urchin models showed a reduction in occupancy in the southern portion of their range under all future climate scenarios when compared to present day, indicating a potential range contraction across RCP scenarios at mid and end-of-century (Figs 2, 3 & S2 Table in S1 File). Giant kelp occupancy was unchanged in the southern region under all RCP scenarios (S3 Table in S1 File) indicating kelp population stability during future climate scenarios, despite thermal stress and expectations for contraction based on patterns in other populations.

**Fig 4:**
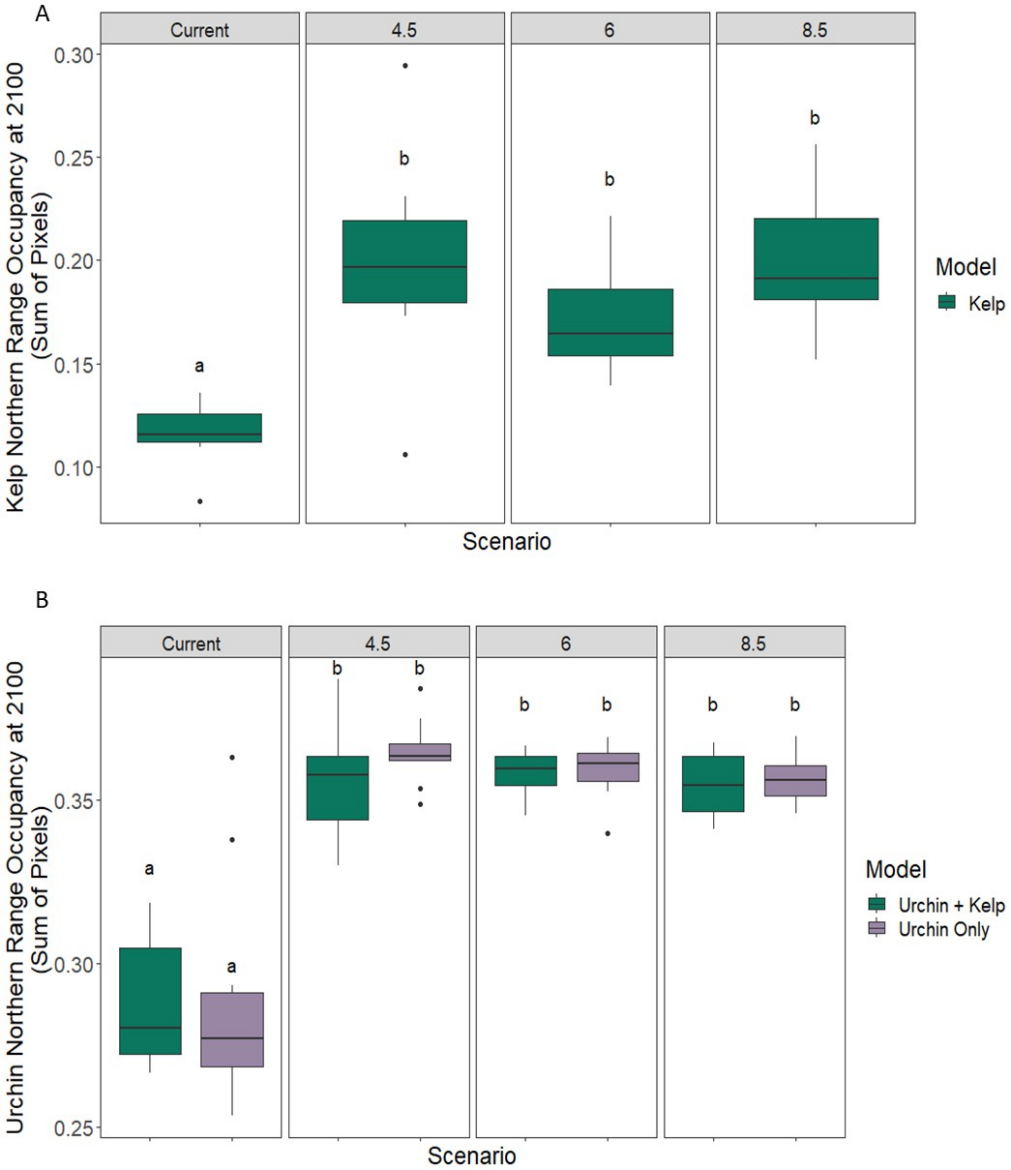
Giant kelp range occupancy. Range occupancy is shown for both (a) kelp and (b) urchins under current day and climate change scenarios in the northern range edge. Kelp and urchins demonstrated potential for a northern range expansion under all future climate scenarios by end of century (Section × Scenario Tukey p-values for kelp were 6.1E-8, 0.0013, and 5.1E-8 for each RCP respectively [S3 Table in S1 File]; Section × Scenario Tukey p-values for urchins were 8.3E-9, 2E-8, and 9.8E-8 for each RCP respectively [S2 Table in S1 File]). See supplement (S4 Fig in S1 File) for mid-century urchin predictions.

**Fig 5:**
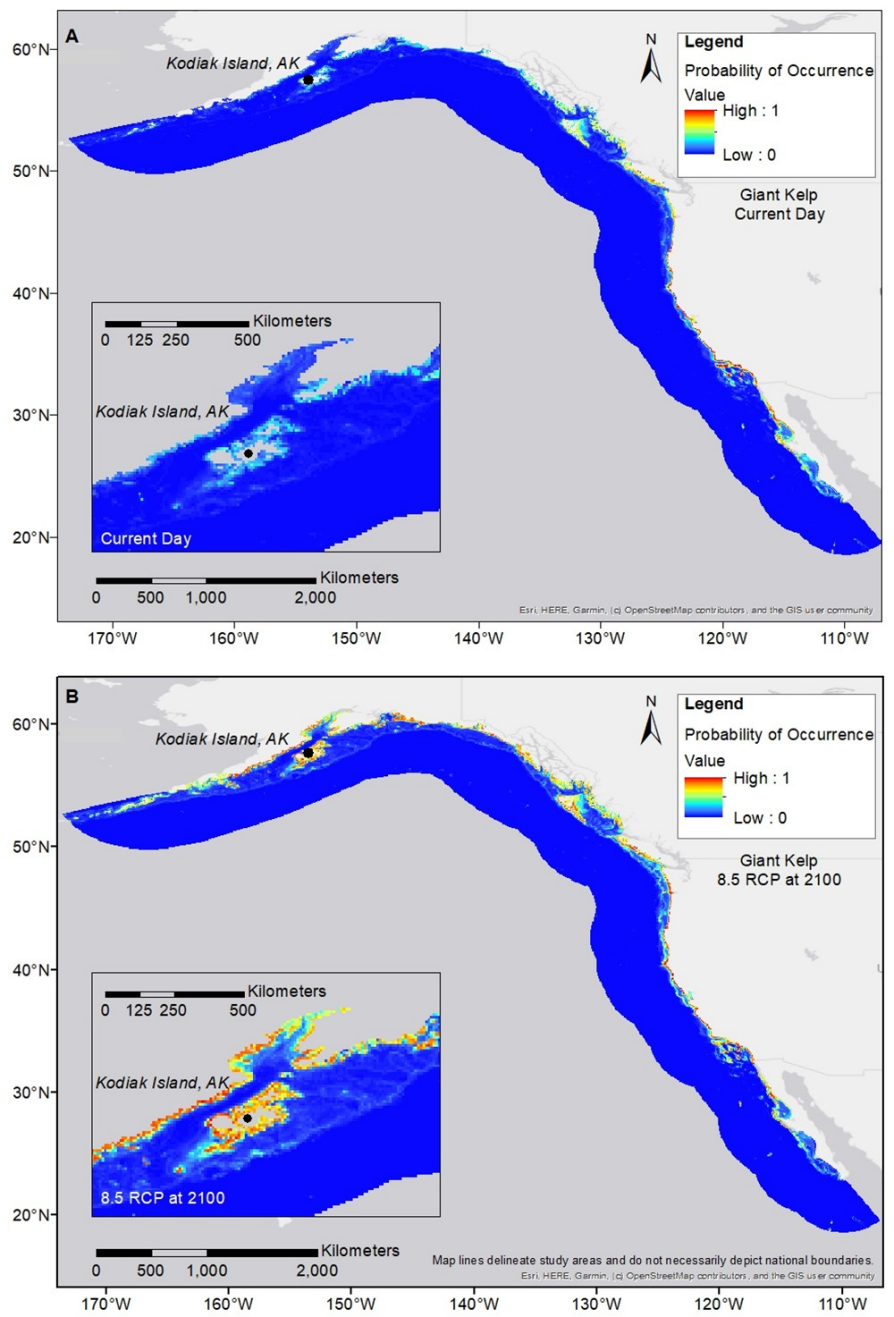
Giant kelp distribution maps. Giant kelp is modeled at RCP 8.5 for end of century; (a) shows the current day projection; (b) the RCP 8.5 scenario for 2100. Pullouts focus on the far northern edge of the species range. Visual increases in geographic spread are seen at the farthest northern edge by end of century.

**Fig 6:**
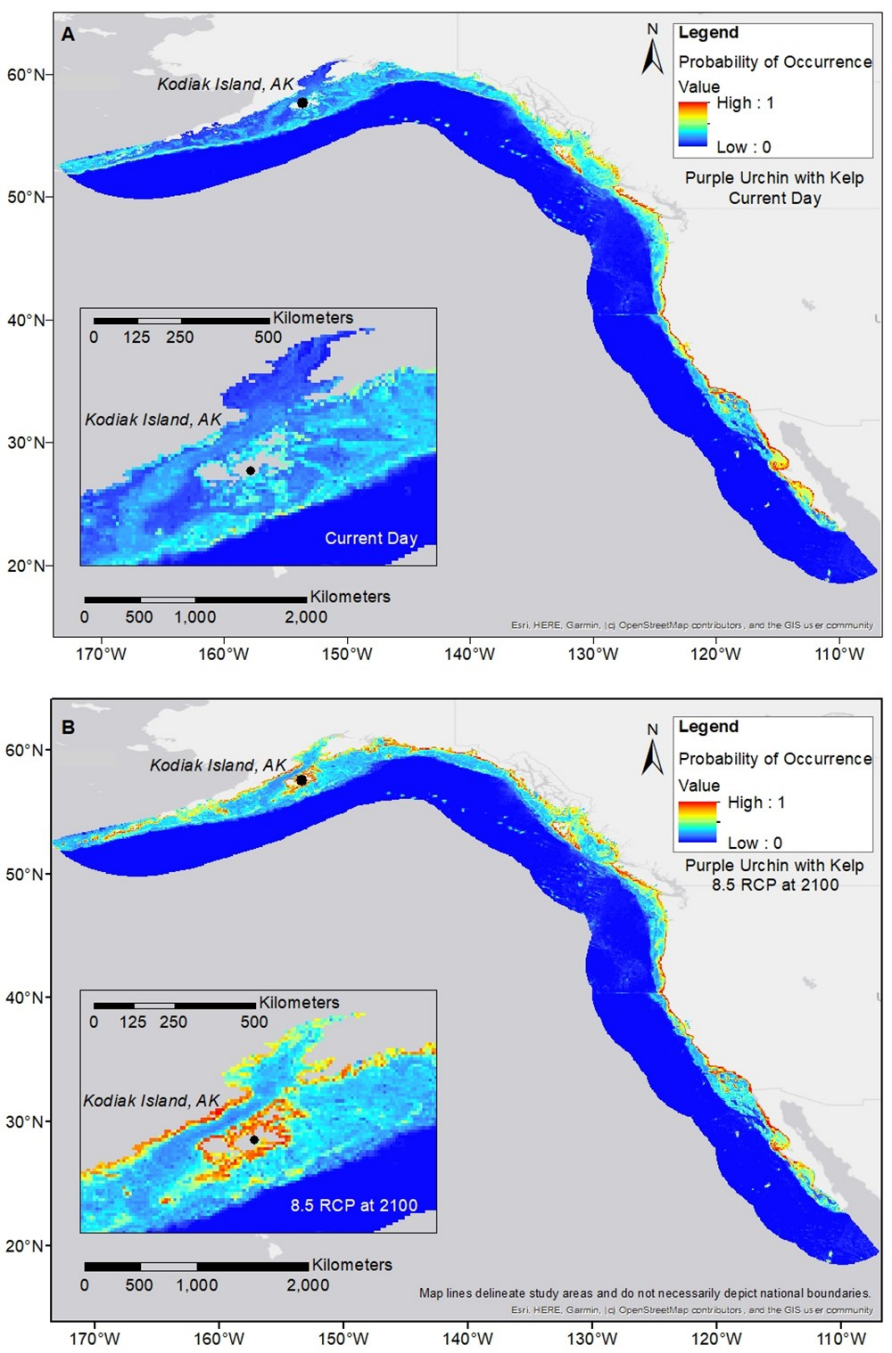
Purple urchin distribution maps. Purple urchin is modeled with giant kelp at RCP 8.5 for end of century; (a) shows the current day projection; (b) shows the RCP 8.5 scenario for 2100. Pullouts focus on the far northern edge of the species range. Visual increases in geographic spread are seen at the farthest northern edge by end of century.

In contrast, climate change is predicted to initiate concurrent poleward range expansion for both purple urchin and giant kelp populations. Giant kelp models showed an increase in occupancy in the north by end of century across RCP scenarios compared to present day (Figs 4A, 5 and S3 Table in S1 File). Models of purple urchin distribution also showed an increase in occupancy in the northern region across RCP scenarios at mid and end-of-century, indicating potential for a northern range expansion of purple urchins that could occur more quickly than for giant kelp (Figs 4B, 6 & S2 Table in S1 File). For both species, the section of range where we expect expansions includes current day marginal habitat. Expansion could include the establishment of more continuous populations in areas where there are currently patchy distributions as well as movement into novel habitat beyond known occurrences. Given that the current distribution of giant kelp already extends further north than the current range of purple urchins, extension of giant kelp range could lay the foundation for further purple urchin expansion in the future. Purple urchin models at current day show a larger range projection than the known current day range indicating that purple urchins are either undersampled at present day, or may not be filling their entire theoretical niche (S1 Fig in S1 File).

In the central portion of purple urchin and giant kelp range there was little projected movement of either species. There was an increase in giant kelp occupancy under the most severe climate predictions (RCP 8.5) during mid-century (S5 Fig and S3 Table in S1 File), but this projection was not retained in the end-of-century models. At end-of-century, there was an increase in giant kelp occupancy under an intermediate climate scenario (RCP 6.0; S6 Fig and S3 Table in S1 File) that was not present at mid-century or in more severe (RCP 8.5) models. Occupancy of purple urchins in the central portion of their range was not predicted to change under any climate scenario (S2 Table in S1 File). Therefore, models predicted more substantial changes to occupancy in the northern and southern portions of both species’ ranges, and relative stability in the central region.

Overall, species distribution models were robust and highlighted the importance of giant kelp and temperature to purple urchin range predictions under climate change. Maxent models had a high degree of predictability with average AUC values that equaled or exceeded 0.95 in all models, and omission rates that were close to the expected values, indicating good model fit relative to random (S4 Table in S1 File). Including giant kelp in the urchin model modified mid-century predictions of range occupancy in the southern section (S3 Fig in S1 File) but did not change the overall pattern of range contraction under climate scenarios, and did not modify results in the central or northern sections of the 2050 model, or in any section of the 2100 model. Giant kelp, however, still had the highest percent contribution to urchin model outputs of all variables (up to 75%) with average temperature being the second highest contributor (up to 38%) for urchin models and highest contributor (up to 60%) for kelp models (S4 Table in S1 File). Maximum temperature was also a strong predictor, contributing 8.8% to giant kelp and 13.9% to urchin-only models. For giant kelp, light at bottom contributed a maximum of 6.6% to model outputs, less than mean and maximum temperature, further emphasizing temperature as a central factor in predicting range shifts under climate change for kelp species. While including giant kelp in urchin models had limited impacts on model results, it did alter the frequency of occupancy. Addition of kelp in the urchin model reduced the number of pixels with higher probability of urchin occurrence and increased the number of pixels with lower probability, giving a similar area of occupancy but a lower probability of occurrence throughout the range (S7 Fig in S1 File).

## Discussion

Climate change is predicted to cause marked and potentially consequential changes to the distribution of purple urchins and giant kelp, with implications for ecosystem stability. While range shifts are occurring poleward around the world, our study emphasizes the nuance of changing species ranges, in particular highlighting non-concurrent range shifts and the potential for mismatches in range for tightly interacting species. These differing movement patterns further emphasize the importance of modeling co-occurring species in tandem, to understand when these mismatches may occur. When movements of primary producers and dominant consumers occur at different rates it creates opportunities for ecological shifts, especially on range edges, facilitating system-wide community dynamics like consumer release [91], changes to competitive networks [92], and altered trophic structures [14].

The southern range edge of the purple urchin is projected to contract without a concurrent reduction in giant kelp occurrence, creating opportunity for a wide variety of community compositional changes. In areas where urchin populations were limiting kelp occurrence through consumption, there is potential for herbivore release [93]. In these situations, the weakening of consumer pressure could allow for an increase in kelp abundance through the re-establishment of historical kelp populations that had been turned into barrens [91], or the facilitation of kelp growth into areas where colonization could not occur due to herbivore pressure on recruits [94]. Urchin culling, the systematic removal of urchins from a barren, can be an effective method for reducing consumer pressure on kelp and facilitating kelp regrowth [91, 93]. These culling events differ significantly from range dynamics but demonstrate that lower urchin abundance can indeed result in kelp recovery. Reduction in urchin densities via range shift could allow for thermally stressed kelp to continue to reproduce and colonize without the two-fold stress of herbivory and changing ocean conditions.

On the northern range edge, where both species could undergo expansion, opportunities could emerge for different types of community change such as novel competition over resources. For giant kelp, northern expansion could lead to spatial competition with other canopy forming species like bull kelp, *Nereocystis luetkeana*. Similarly, as urchin populations move northwards competitive dynamics could unfold with other resident urchin species. Red urchins, *Mesocentrotus franciscanus*, which currently have a range boundary further north than purple urchins, could experience potential competition for space and other resources. While in their current range there is debate about the interaction of red and purple urchins, with some evidence of competition and facilitation [95], their current niches are thought to differentiate along gradients of depth and wave action [96]. The movement of purple urchins into areas where red urchin populations already occur could lead to competition for preferred habitats, especially in areas where other urchin populations, like green urchins, *Strongylocentrotus droebachiensis*, also co-occur. Competitive interactions among these kelp and urchin species occur in more southern portions of the giant kelp and purple urchin ranges in the Eastern Pacific but would be new for the far northern range undergoing novel expansion, with outcomes that may be difficult to predict.

Adding to the complexity of northern community compositional change, areas of projected expansion of both species include both novel habitat and patchy marginal habitat. These areas of marginal habitat include a network of patches, where occurrences have been observed, followed by large gaps where occurrences have yet to be noted. In some cases, these patches may have established populations, but in other cases they may be singular observations. Generally, marginal habitat is well represented by areas of low probability of occurrence in current day range projections (S1 & S2 Figs in S1 File). Expansion may include new occupancy of truly novel habitat, but it may also include the filling in of marginal habitat with more continuous populations. Projected expansions will likely include both types of expansion occurring at different time scales as populations move. Further, these kinds of species distribution models, while widely used to make long-term predictions based on projected changes to climate means, are silent on how these changes may unfold over time given high variability in many marine systems [97] and the likelihood that acute warming or cooling events may expedite or dampen these changes at shorter timescales than represented here. Modeling across large geographic scales sacrifices the intricacies of community specific change or short-term projections for a better understanding of ecosystem wide population drivers that are equally as valuable to understanding the impacts of global change.

**Fig 7:**
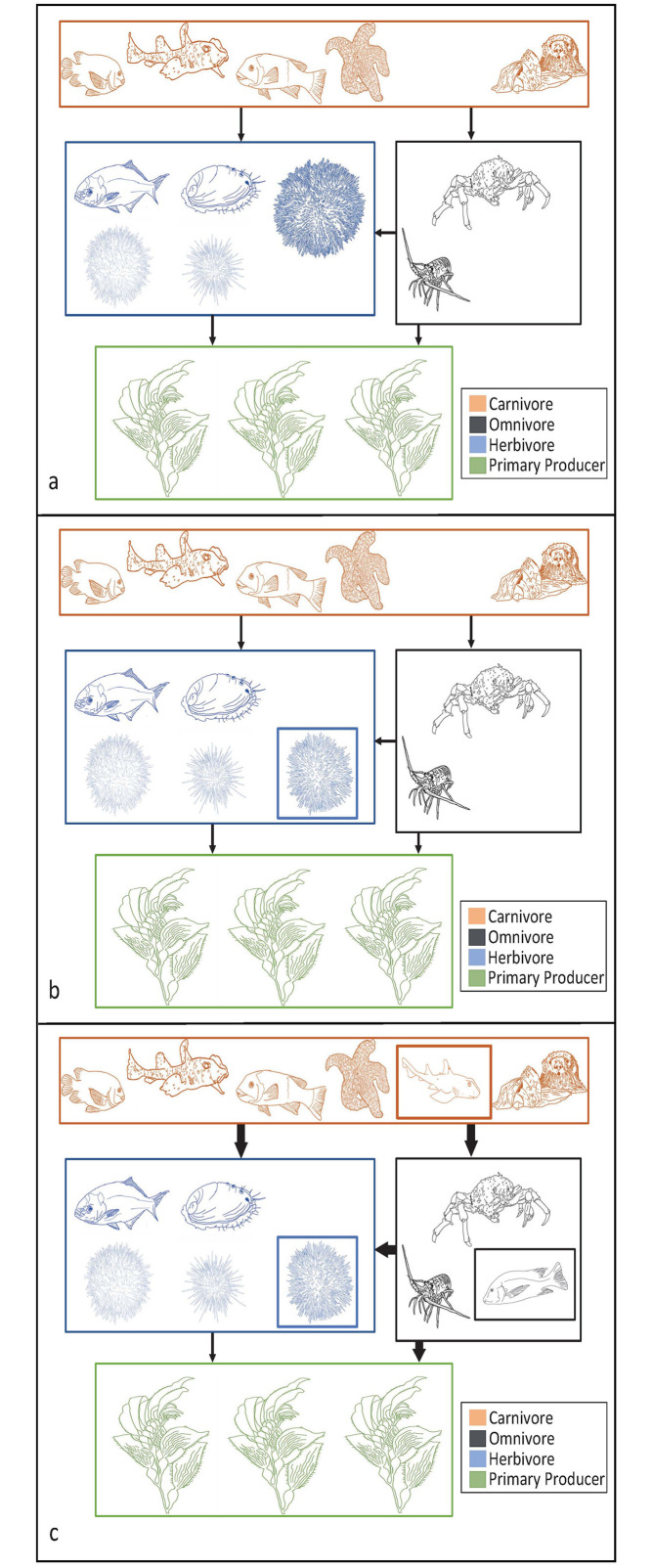
Conceptual southern food webs. Conceptual diagrams of simplified food webs for the southern range edge of purple urchins and giant kelp, demonstrating both the modeled contraction of purple urchins and hypothesized community dynamics that may result from this change. (a) Current day food web with the purple urchin represented in the upper right corner of the herbivore box, and the giant kelp represented in the primary producer box. (b) Southern range food web with the modeled removal of purple urchins and the addition of another urchin species filling the abandoned niche shown by the blue box. Interaction strength between groups, as indicated by arrow widths, is expected to remain stable due to the continuity in number of species per trophic group. (c) Southern food web with purple urchin removed. This niche is filled by another urchin species shown in the blue box as in panel (b). In addition, hypothesized tropicalization could result from the movement of other tropical generalist species moving into the range, shown by the black and orange boxes. The addition of these species could increase consumer pressure (thicker arrows) on lower trophic levels due to the increase of consumer species. Please see S5 Table in S1 File for species represented in the diagram. All drawings are original by M. Cortese.

**Fig 8:**
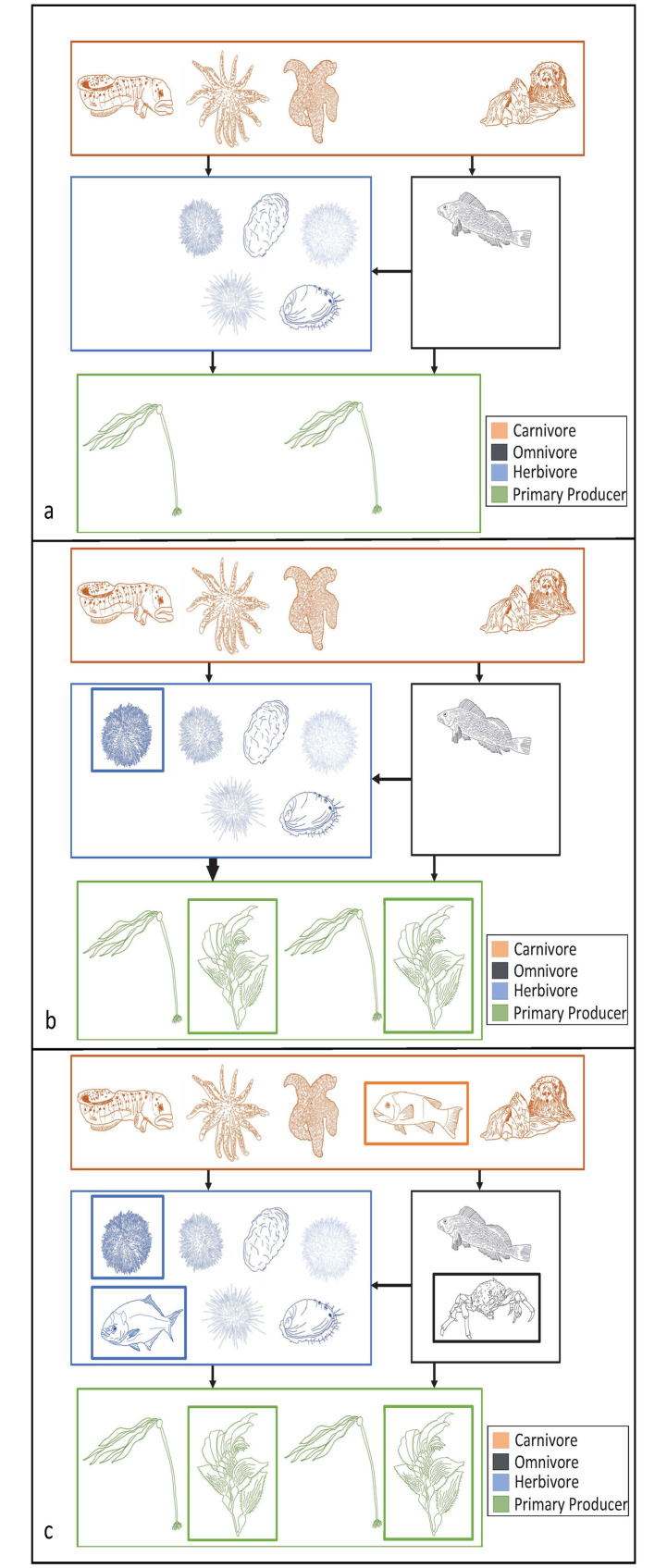
Conceptual northern food webs. Conceptual diagrams of simplified food webs for the northern range edge of purple urchins and giant kelp, demonstrating both the modeled expansion of these species into current day marginal habitat, and the hypothesized community dynamics that may result from these changes. (a) Current day food web for the northern range edge where both purple urchins and giant kelp are rare. (b) Northern edge food web with the modeled addition of purple urchin and giant kelp, shown by the new species in the herbivore and primary producer boxes. The addition of these species could increase competition among species in the same trophic level due to resource limitations. The addition of another mid-trophic level herbivore may increase consumer pressure on producers (thicker arrows). (c) Far northern edge food web with the modeled addition of purple urchin and giant kelp as in panel (b). In addition, theorized range shifts of other generalist consumers could allow for concurrent expansion, shown by the addition of species highlighted with black, blue, and orange inner boxes. The movement of both mid and high trophic level consumers may lead to food web stability, reducing the herbivore pressure shown in panel (b) by increasing higher level consumer pressure. Please see S5 Table in S1 File for species represented in the diagram. All drawings are original by M. Cortese.

While models in this study only provide evidence for movement of purple urchin and giant kelp, it is important to consider these shifts in a larger context of community interactions (Figs 7 and 8). Although the continued stabilization of southern kelp forests sounds promising, the removal of dominant herbivores from the southern range edge creates a niche for another more tropical herbivore to take its place. Topicalization has been occurring throughout temperate zones, where more tropical species have been moving into warming temperate regions to avoid heat stress at lower latitudes [98]. Movement of temperate species poleward may further facilitate the colonization of novel tropical herbivores into the community. Tropical herbivorous fish, for example, can move into temperate areas due to their high mobility and generalist consumer strategy [17]. In Australian kelp forests, an increase in herbivorous fish richness due to ocean warming has been correlated to an increase in kelp forest herbivory and a decline in kelp [99], demonstrating that novel consumers may continue to compound stress on kelp. Other more tropical urchin species could also fill the niche of purple urchins. In particular, the low-lying sea urchin or white sea urchin, *Tripneustes depressus*, which is known to consume macroalgae and already occupies parts of Baja California, could be a possible candidate for filling the abandoned niche (Fig 7b). Additionally, traits such as trophic level (omnivores), motility (swimmers), and latitudinal range size (larger range) make species more likely to undergo range expansion [100] indicating that in addition to herbivorous fish, we may also see range shifts in motile higher trophic level omnivorous species. The addition of these more generalist species could increase consumer pressure on mid trophic species (Fig 7c).

On the northern range edge (Fig 8), movement of purple urchins into an area where they do not have consistent established populations could lead to increased consumer pressure on kelp (Fig 8b). Establishment of kelp species into currently patchy habitat could lead to spatial competition over substrate between macro-algae species (Fig 8b). Spatial competition combined with increased herbivory from urchins could compound stress on kelp. In addition to the movement of purple urchins, other more southern temperate consumer species may also shift their ranges north. Herbivorous fish as well as generalist omnivores and carnivores like the sheep crab or California sheepshead have traits that make them more likely to expand poleward, such as omnivorous diets, adult motility, and large latitudinal ranges [100]. These movements paired with the urchin and kelp movement may stabilize food web structure by expanding the composition of multiple trophic levels simultaneously (Fig 8c).

This study highlights the ecological impacts of range shifts, from changes in food web structure to altered interactions. Not only are range edges more at risk for species geographic movement, but also the subsequent changes in community interactions that come with species range shifts. When tightly paired species move at different rates, the resulting impacts can also transcend ecological scales. Modeling co-occurring species in tandem has provided insight into different ways the community may change. While these insights are valuable there are still ways to continue to build more ecologically relevant projections. These models are based on presence only data, a common method that makes modeling across such large spatial scales possible [101]. These models show changes to species presence and absence across large spatial scales, but do not make explicit predictions about changing abundances despite the close connection between occupancy and abundance. Changing abundance patterns could impact community interactions across the species range. Kelp populations, which were predicted to decline but remained stable across modeling scenarios, may still experience decreases in abundance that were not detected by large-scale presence/absence models. Valuable insights could be gained by attempting to model sections of these species’ populations using abundance data to better parse out shifting abundance and changing interactions among species. Modeling this community at different scales could also provide insights into which factors drive the biogeography of species at a community level, a population level, and a species level. This study, with the full species range models, makes necessary strides in understanding species and community wide drivers of change, but for drivers of smaller scale population dynamics, further information is needed. Additionally, we know that beyond geographic changes, altered species interactions will also occur under warming due to changes in metabolism [102, 103]. While this study does not quantify changes in interaction strength, distribution modeling provides insights into where changes to interaction strength might be most impactful on community structure.

Species distribution models are commonly used to better understand species ranges. While these models are robust, there are some caveats in using these modeling frameworks. Despite Maxent’s ability to operate well with highly correlated environment variables [56], overfitting can occur. Additionally, the multi-layered nature of these models adds an unknown level of uncertainty to model outputs. Uncertainty for these models has multiple sources including the use of citizen science data, development of environmental layers based on RCP projections, and the integration of kelp projections into urchin + kelp models. Distribution models are often subject to these types of concerns. We have attempted to reduce uncertainty when possible, and our models have high AUC values and omission rates within the expected range for well-fitting models. Our projections, therefore, provide strong data-driven estimates that can inform ecological predictions as well as management decisions related to conserving ecosystem functions into the future.

Changes to geographic range for ecologically important species such as giant kelp and purple urchin could have widespread impacts starting with their immediate ecological communities, spreading to industries that are dependent on healthy kelp forests along the coast such as fisheries and tourism, and even reaching ecosystem services like coastal erosion management. Range shifts can cause substantial impacts on management and industry in marine systems more broadly [19], and such impacts are likely to emerge in this system as well. For these species, altered range could cause community compositional change which could then impact fisheries of purple urchin and of other herbivorous fish as they move to fill open niches. Southern range contraction for urchins could improve management efforts to restore urchin-barrens and stabilize kelp populations in areas where there have been phase shifts. In contrast, the potential addition of more tropical urchin species, via tropicalization, to an area already undergoing over-grazing could reverse urchin removal efforts and further stress kelp populations. Additionally, range shifts in these species and subsequent community compositional changes could impact kelp forest tourism related to SCUBA, specialty fishing, and wildlife viewing. By continuing to model closely interacting species together, we can better understand these differences in movement patterns and better prepare for ecosystem conservation rather than single species management.

## Supporting information

S1 File (DOCX)
