# Supplementary material for: When species don’t move together: Non-concurrent range shifts in Eastern Pacific kelp forest communities
Source: PLoS One. 2024 May 24;19(5):e0303536. doi: 10.1371/journal.pone.0303536 (PMC11125554; doi:10.1371/journal.pone.0303536)
Supplement: S1 File — (DOCX) [file pone.0303536.s001.docx]

When species don’t move together: non-concurrent range shifts in eastern pacific kelp forest communities

Mary R. Cortese, Amy L. Freestone

Supplementary:

Table S1: Model variables and definitions.


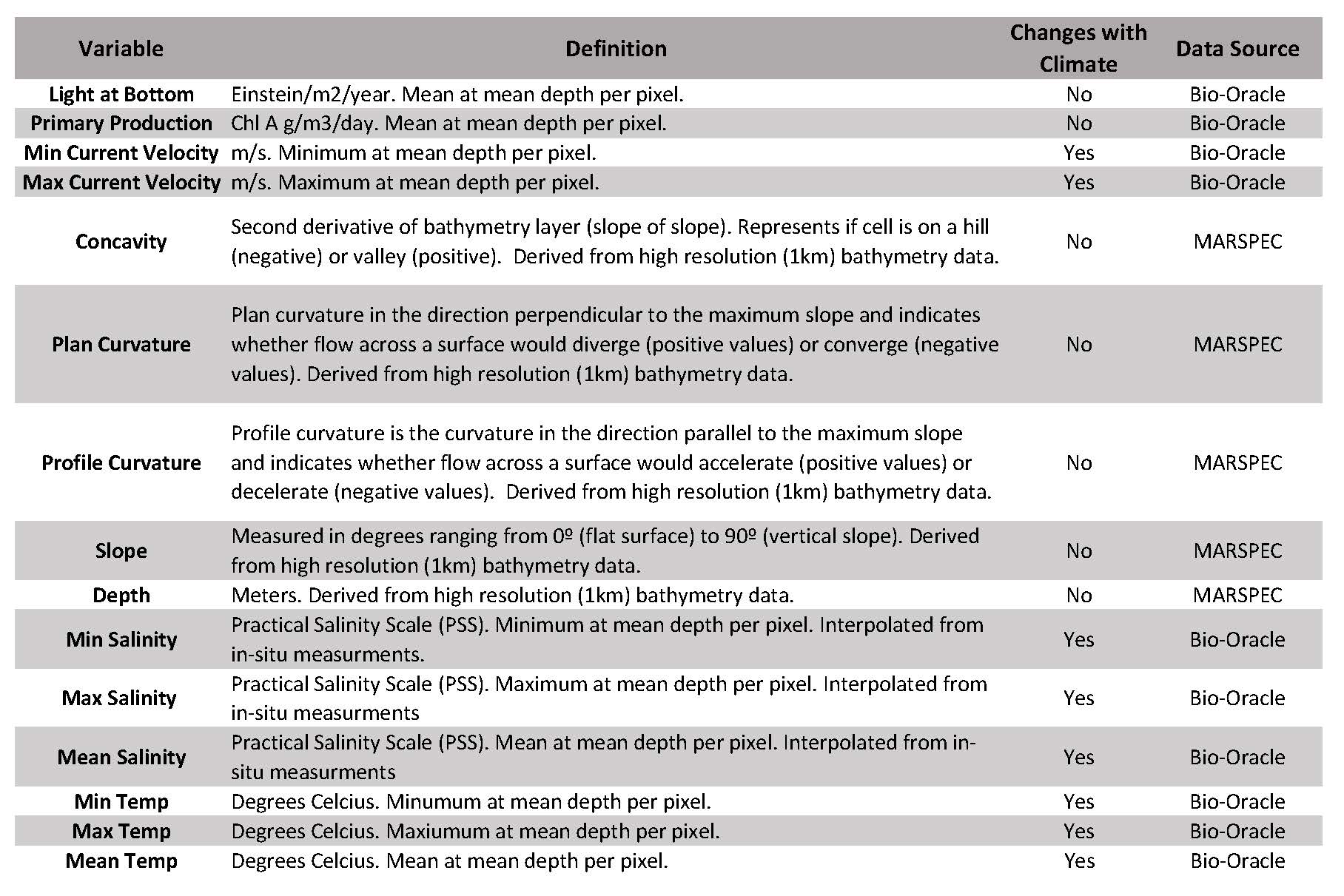


Table S2: Outputs for 2050 and 2100 GLM and Tukey means comparisons of urchin range occupancy. Significant comparisons are highlighted in gray. For 2050, there were notable increases in urchin range occupancy across RCPs in the northern range section as well as reductions in urchin range occupancy across RCPs in the southern range section. Additionally, there is a difference between urchin models (urchin + kelp and urchin only) in the southern range section. For 2100, there were notable increases in urchin range occupancy across RCPs in the northern range section and reductions in the southern range edge. Interactions not listed (Species × Scenario, Species × Scenario × Section) were not significant in the GLM. For 2100, Species × Section was not significant in the GLM so Tukey results are not reported.
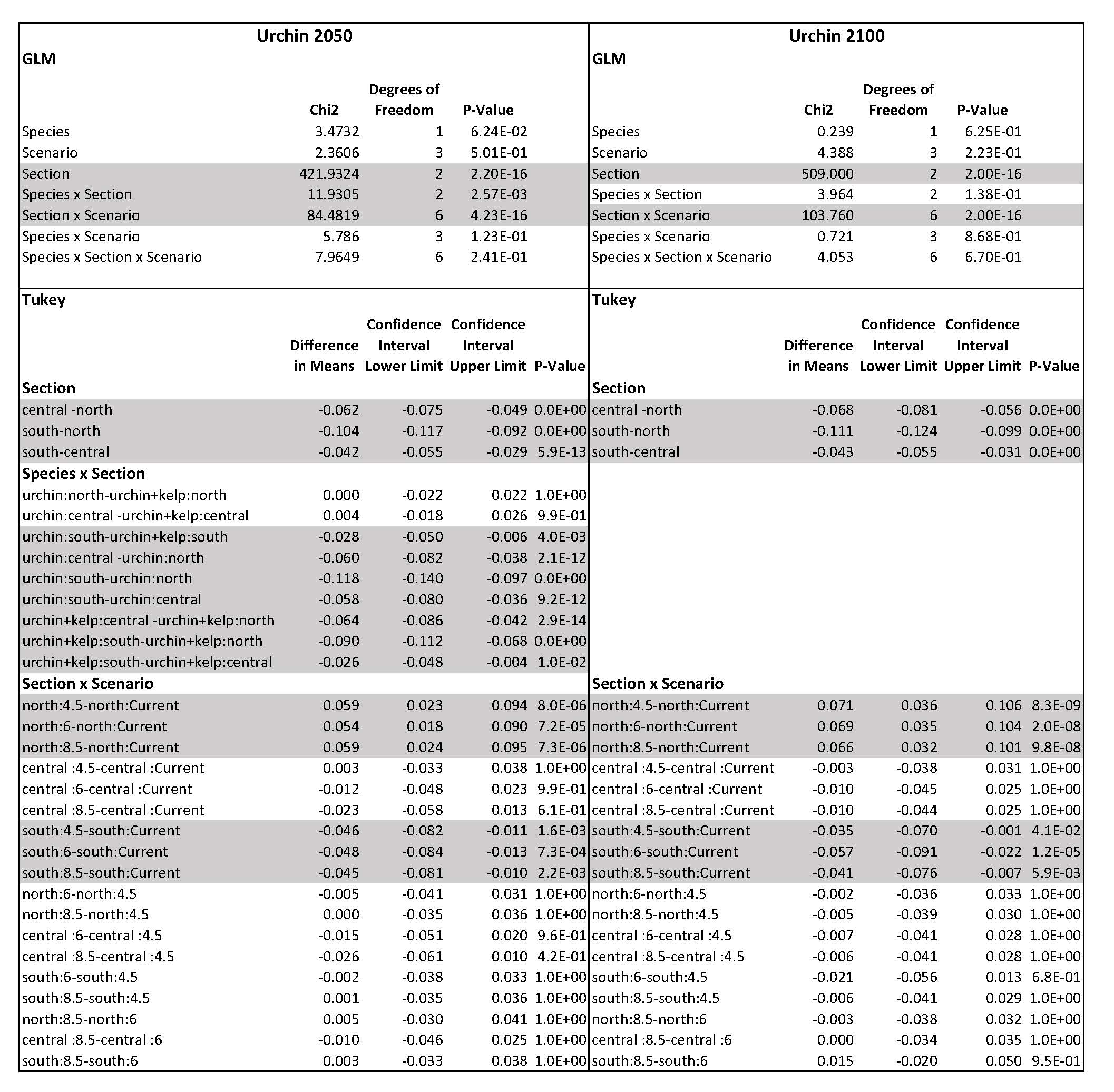


Table S3: Outputs for 2050 and 2100 GLM and Tukey means comparisons of kelp range occupancy. Significant comparisons are highlighted in gray, notably an increase in kelp range occupancy in the 2050 central section under RCP 8.5 and an increase in kelp range occupancy in the 2100 northern section across all RCPs and in the central section under RCP 6.0.
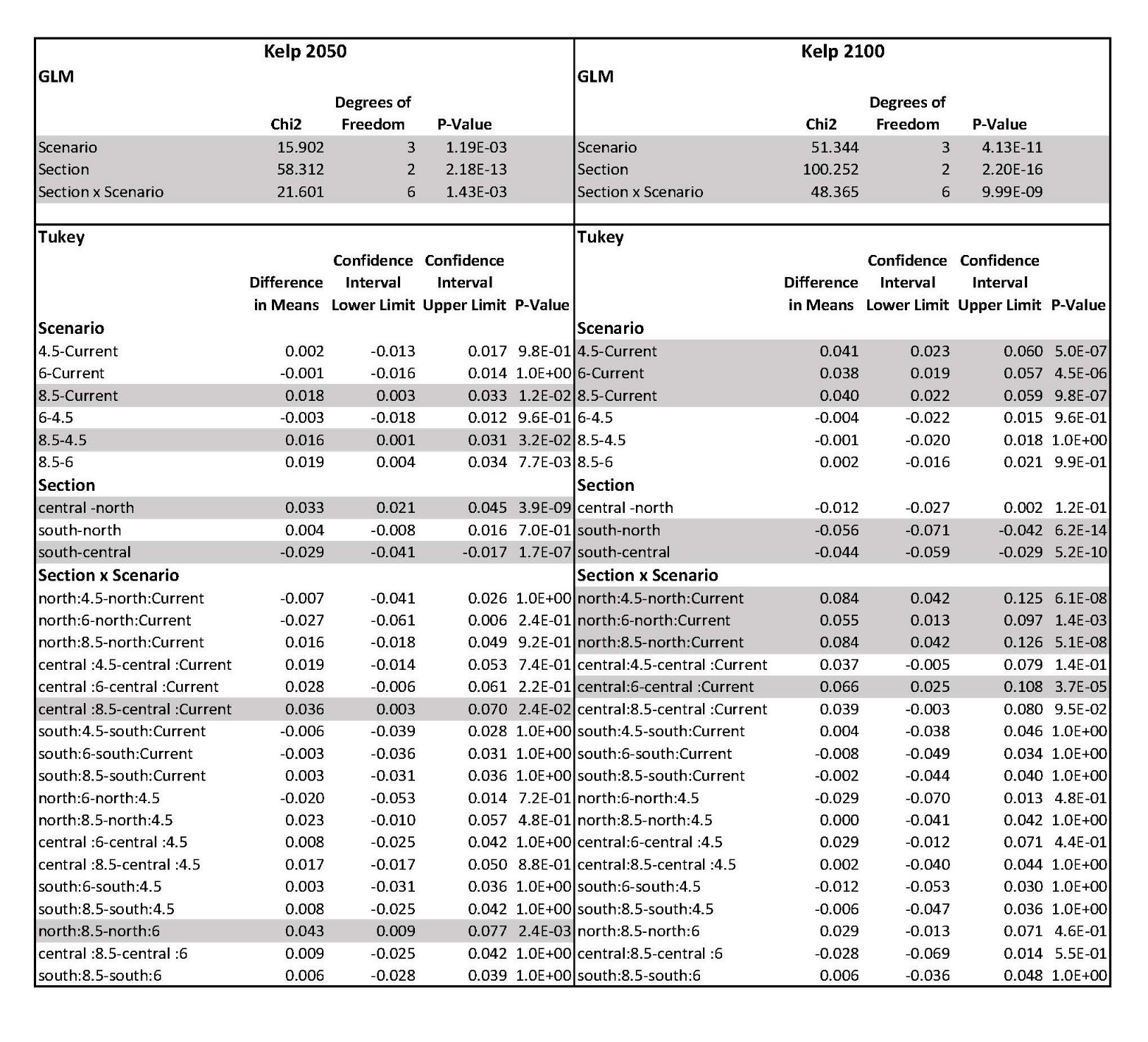


Table S4: Model AUCs, omission rates, and variable contributions to model outputs.


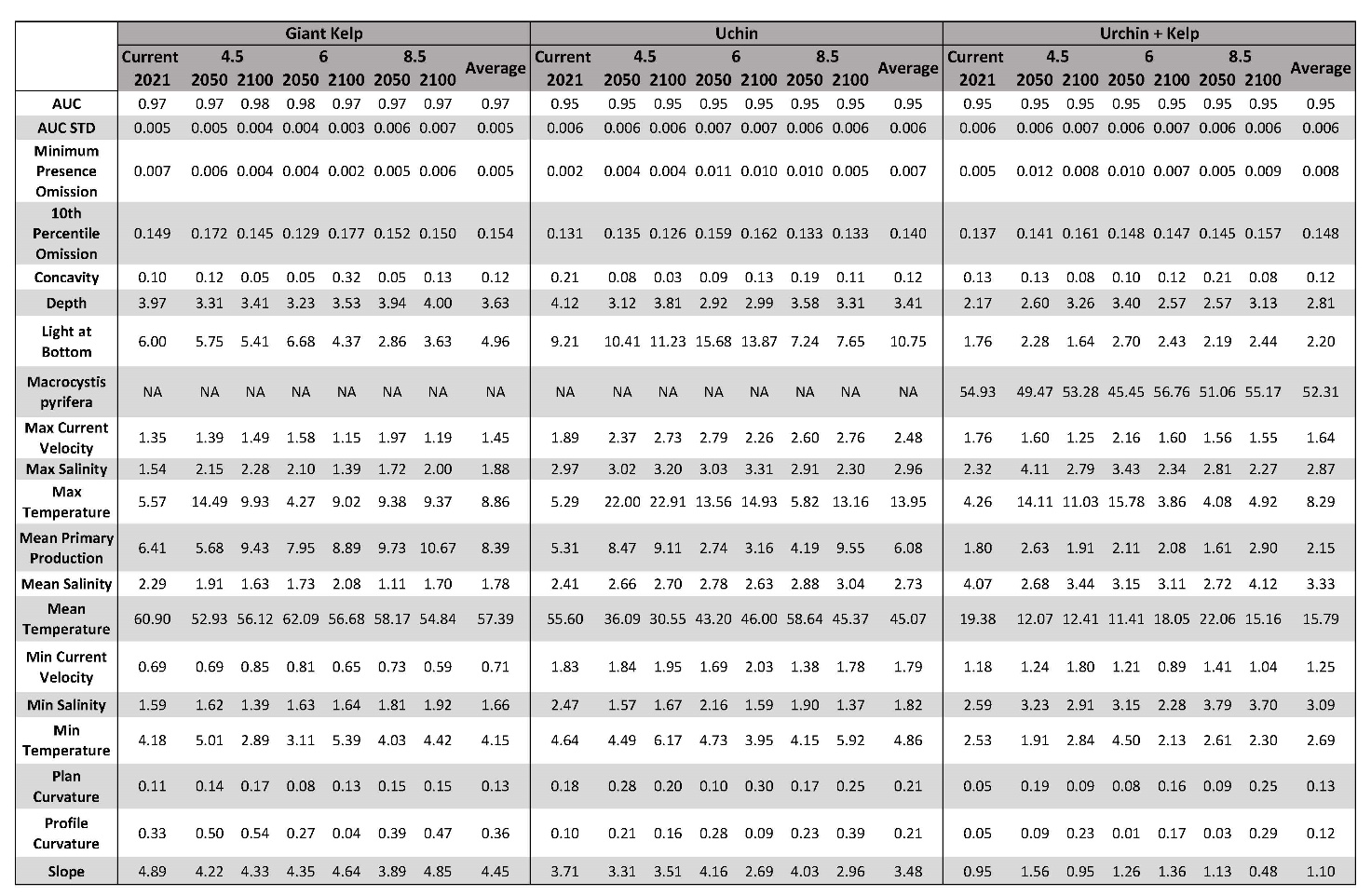


Table S5: Food web species, grouped by range edge.


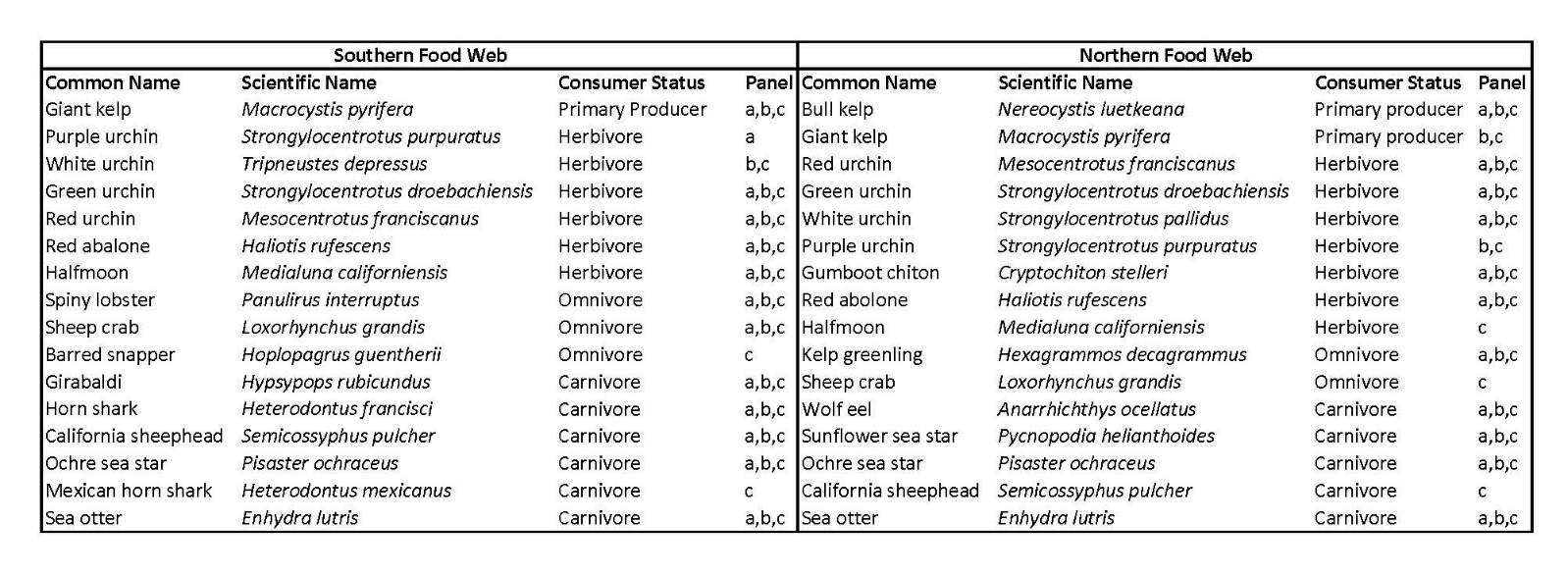


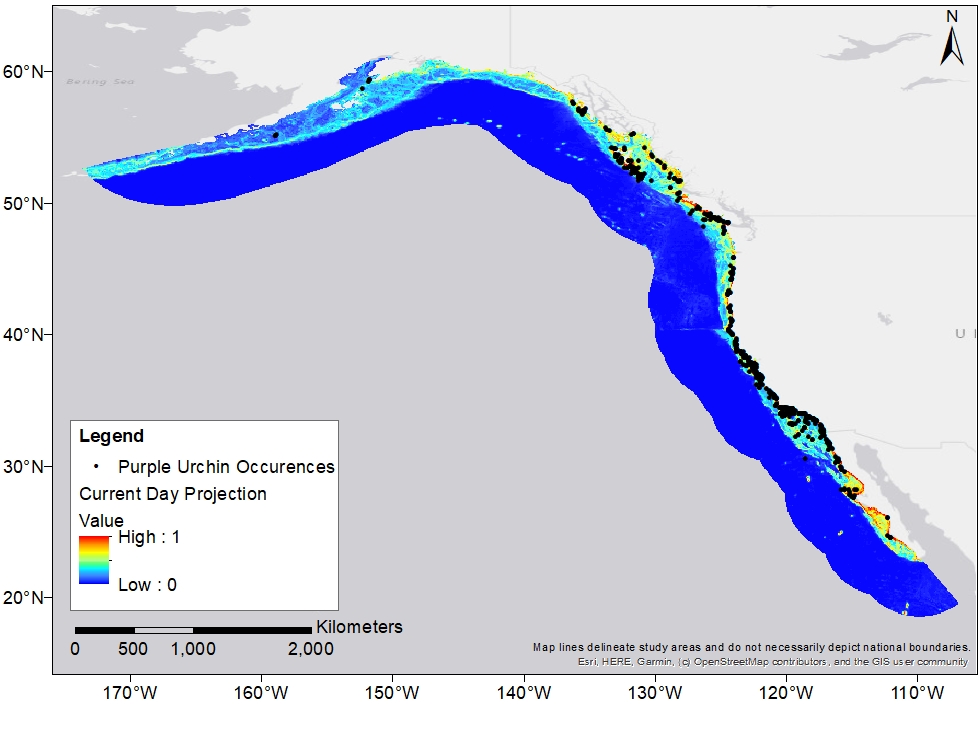


Figure S1: Purple urchin occurrence records used in Maxent models, overlayed on current day Maxent range projection.


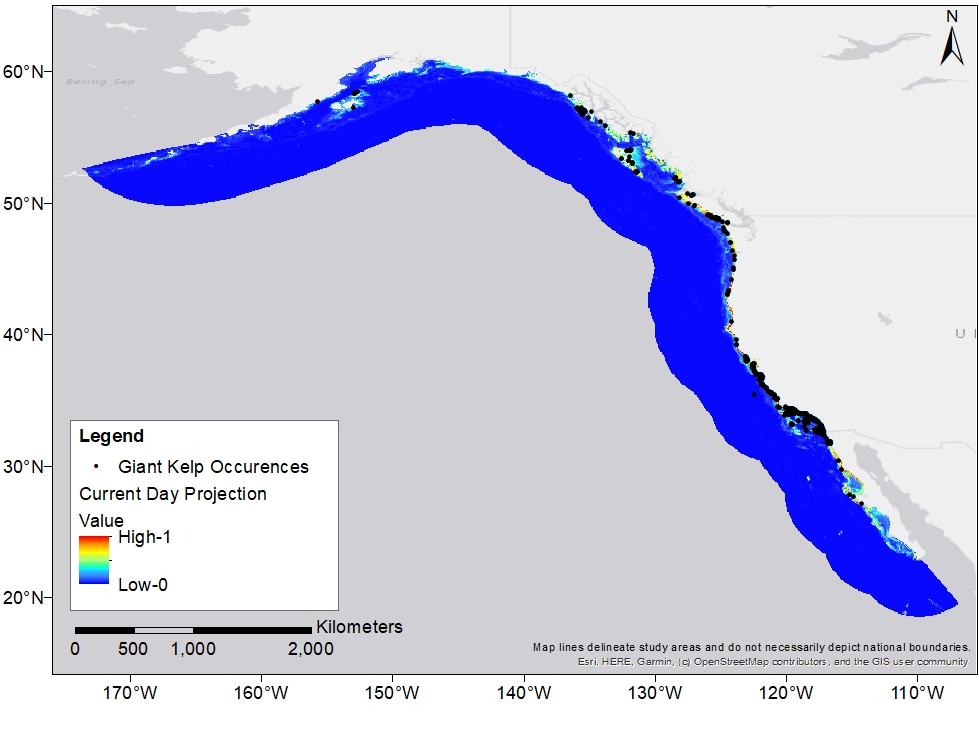


Figure S2: Giant kelp occurrence records used in Maxent models, overlayed on current day range projection.


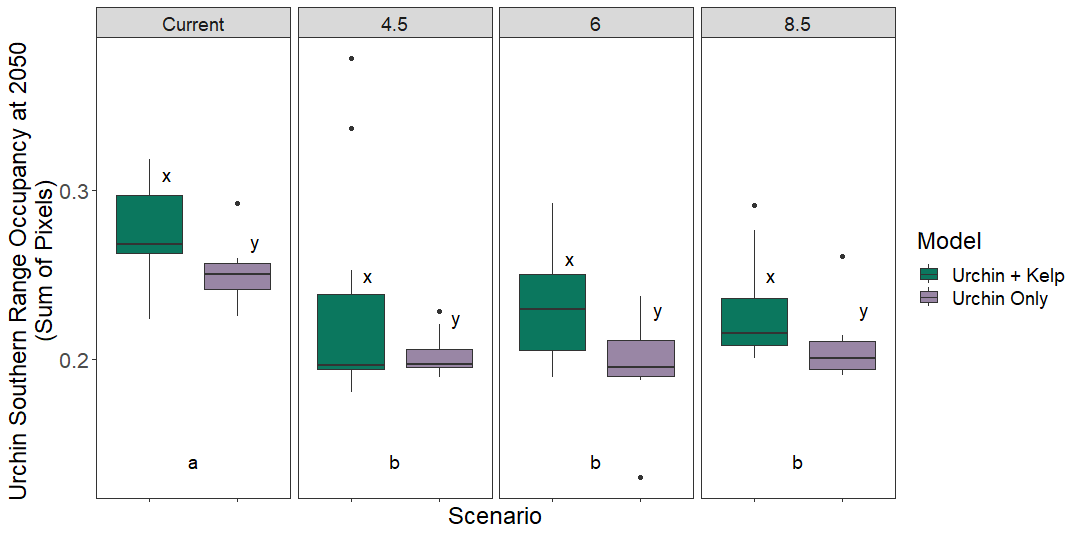


Figure S3: Purple urchins are predicted to experience a range contraction in the southern portion of their range under all mid-century climate change scenarios, with less range occupied in urchin only models (purple) rather than with kelp included as a model predictor (green) across all scenarios including current day. Predicted area of occupancy in the southern range are shown for current day and mid-century climate projections. There are reductions in range occupancy in all scenarios when compared to the current day (letters a + b; Section × Scenario Tukey, Table S2). While kelp inclusion did moderate reductions in predicted urchin range occupancy (letters x + y; Species × Section Tukey Table S2), this pattern was not seen in end of century models or at other range sections. There was no significant three way interaction between Species ×Section × Scenario.


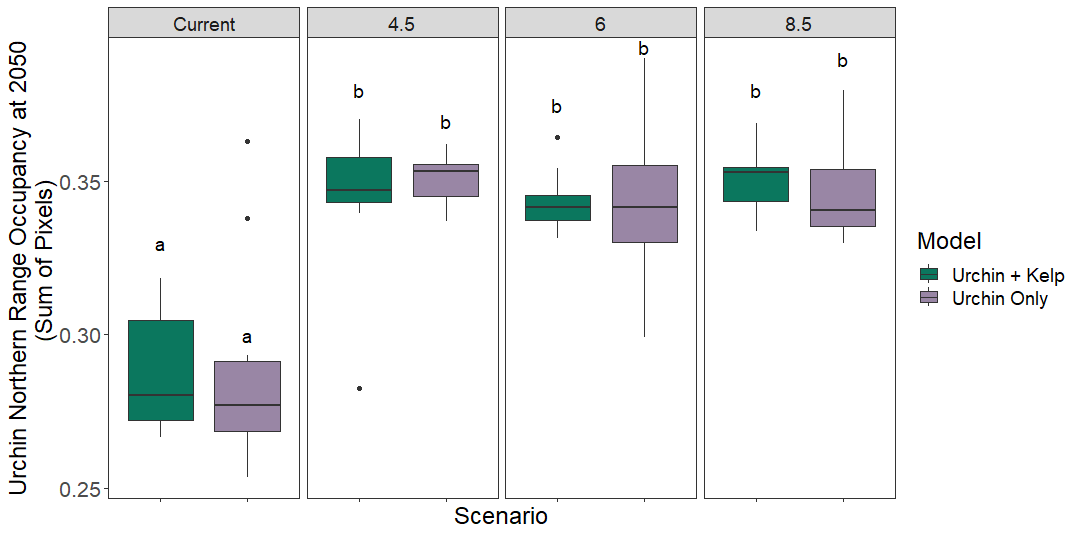


Figure S4: Purple urchin are predicted to undergo range expansion in the northern section of their range under mid-century climate predictions. Predicted area of occupancy in the northern range are shown for current day and mid-century climate projections. There are increases to range occupancy in all scenarios when compared to the current day. Results were similar when urchins were modeled with only environmental conditions or when using kelp as an additional predictor variable. See Section × Scenario Tukey in Table S2.


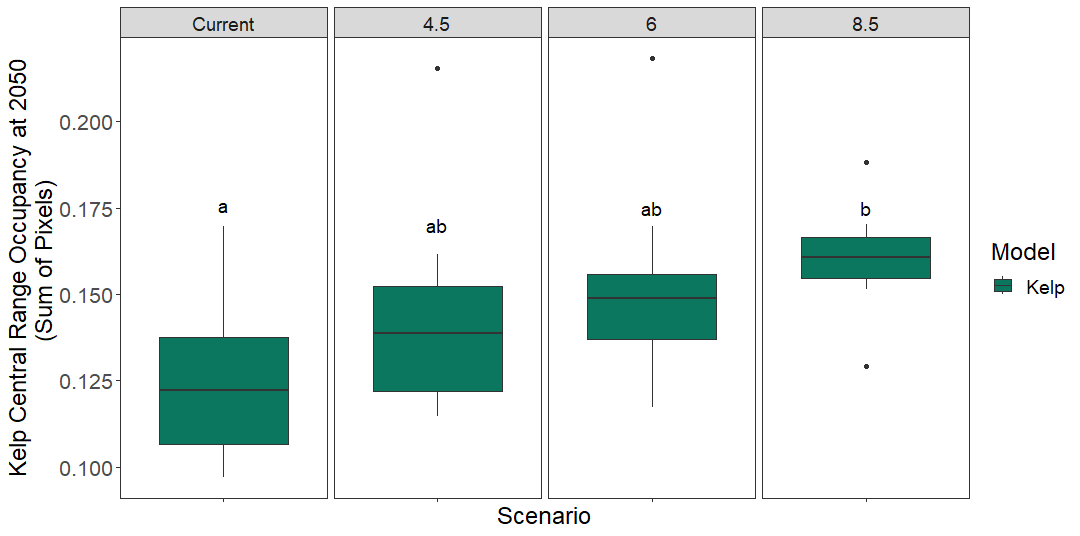


Figure S5: Giant kelp are predicted to undergo range expansion in the central section of their range under some mid-century climate predictions. Predicted area of occupancy in the central range are shown for current day and mid-century climate projections. There are increases to range occupancy at the worst case (8.5 RCP) scenario when compared to the current day. See Section × Scenario Tukey in Table S3.


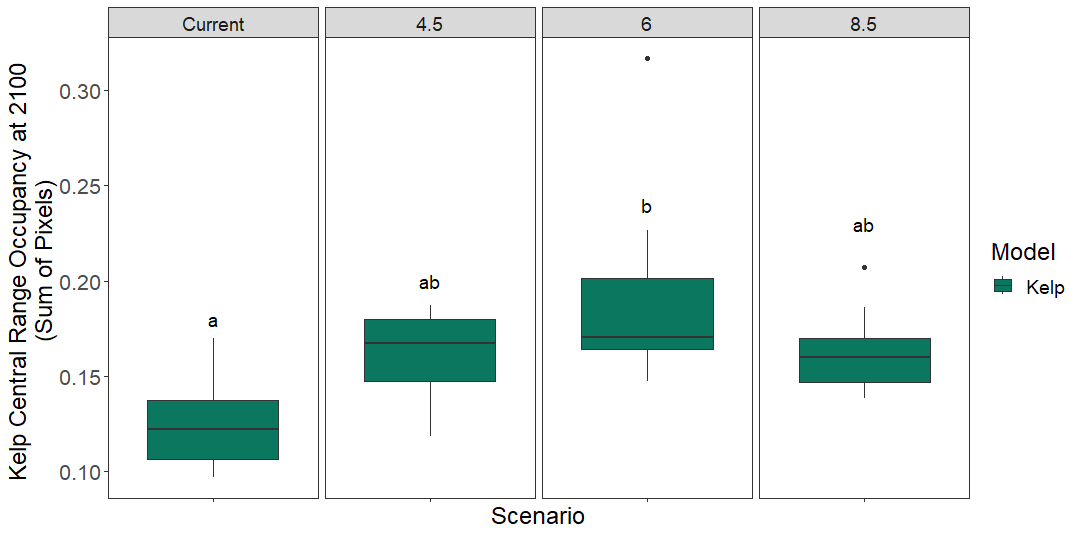


Figure S6: Giant kelp are predicted to undergo range expansion in the central section of their range under some end-of-century climate predictions. Predicted area of occupancy in the northern range are shown for current day and end-of-century climate projections. There are increases to range occupancy at the moderate case (6.0 RCP) scenarios when compared to the current day. See Section × Scenario Tukey in Table S3.


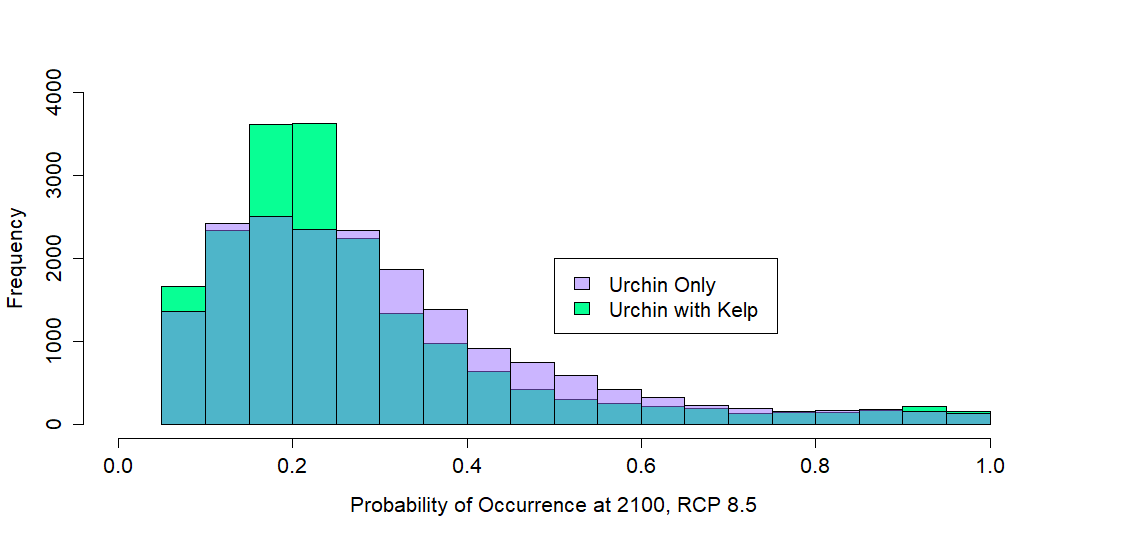


Figure S7: Distribution of the probability of occurrence across species range for both urchin models. The addition of kelp to models reduced the number of pixels with higher probabilities (30-70% chance of occurrence) and increased the number of pixels with lower probabilities (5-25% chance of occurrence), demonstrating how the addition of kelp altered urchin models without changing the overall area of species distribution based on the bounding thresholds.
